# Study on the effects of ecological cognition and organizational support on the willingness and behavior of farmers in arid areas to participate in shelterbelt management

**DOI:** 10.1038/s41598-026-56272-4

**Published:** 2026-06-05

**Authors:** Huijuan Du, Yujiang Yan, Juan Yao, Yuanyuan Xiong, Yuezhao Duan, Hua Tang

**Affiliations:** College of Economics and Management, Xinjiang Agricultural University, Ürümqi, 830052 China

**Keywords:** Farmland shelterbelts, Ecological cognition, Organizational support, Willingness and behavior to participate, Ecology, Ecology, Environmental social sciences

## Abstract

Farmland shelterbelts serve as a crucial ecological barrier for agricultural production operations in ecologically fragile rural areas. Farmers’ participation in the management and protection of these shelterbelts is a key prerequisite for ensuring the sustained and effective realization of their ecological benefits. Based on the social-ecological system framework and interactive determinism theory, this study focuses on 322 farming households in Wensu County, Xinjiang, and employs a bivariate Probit model to explore the effects of ecological cognition and organizational support on farmers’ willingness and behavior to participate in shelterbelt management, as well as generational differences. The findings indicate that: (1) Within ecological cognition, both ecological knowledge and ecological responsibility significantly promote farmers’ willingness and participation behavior; however, ecological values have a negative impact on some participation willingness and behavior due to farmers’ perception of economic losses from the shelterbelt “negative edge effect.” (2) In terms of organizational support, both instrumental support and emotional support significantly and positively influence farmers’ willingness and participation behavior. (3) Organizational support partially moderates ecological cognition. However, neither type of support can mitigate the negative impact of ecological values. (4) Regarding generational differences, younger farmers are more influenced by ecological knowledge and instrumental support, whereas older generations rely more on ecological responsibility and instrumental support. Based on these results, this paper recommends strengthening the cultivation of farmers’ ecological cognition, implementing multiple measures to alleviate the negative edge effect of shelterbelts, and improving a dual-dimensional organizational support mechanism combining emotional and instrumental support to promote the long-term management and protection of shelterbelts.

## Introduction

Farmland shelterbelts serve as vital ecological barriers, ensuring high and stable agricultural yields, optimizing regional ecological security, and promoting the coordinated development of ecology and economy^1^. Since the implementation of the Three-North Shelterbelt Program in 1978, extensive farmland shelterbelts have been established across China’s northwest, north, and northeast regions, forming a composite farmland shelterbelt system designed to maximize multidimensional benefits, optimize regional ecological environments, and improve agricultural production conditions^2,3^.The Tarim Basin region in southern Xinjiang has emerged as a key production area for China’s specialty forest fruits. Surrounded by deserts, its fragile ecological environment has fostered an agroforestry system integrating “ecological forests (shelterbelts) and economic forests (fruit trees).” Shelterbelts provide a stable environment for fruit tree growth through ecological functions such as windbreak and sand fixation, as well as local climate regulation. Fruit tree cultivation not only has ecological value but also generates economic benefits that support the construction and maintenance of shelterbelts, creating a synergistic effect in which ecological and economic benefits mutually reinforce each other. The health and stability of shelterbelts are prerequisites for the sustainable operation of this agroforestry system. The long-term release of their ecological benefits relies on routine management and maintenance measures^4^. As the core beneficiaries of both ecological and economic benefits within this system, farmers’ interests are interdependent on the overall functionality of the system. Due to factors such as the large scale of investment in shelterbelt construction and unclear property rights demarcation, their initial supply is primarily funded through government-organized financing, while subsequent maintenance largely relies on cooperation among farmers^5^. Therefore, coordinated management of both forest types by farmers is crucial for ensuring the sustained protective functions of shelterbelts and promoting the synergistic provision of ecological and economic products within the composite system. However, influenced by inherent characteristics such as the “externalities” and “negative edge effect” of farmland shelterbelts, coupled with farmers’ rational economic behavior that prioritizes economically productive forests over protective ones, management of shelterbelts is often neglected. Under Xinjiang’s unique geographical and climatic conditions, the management of shelterbelts is particularly crucial^6^. This management imbalance has led to severe degradation of shelterbelts, including gaps and breaks in the shelterbelt network, poor tree growth, and diminished ecological functions, significantly constraining the overall benefits and sustainability of the composite system^7–10^.

Farmers are both beneficiaries of the ecological benefits of shelterbelts and key actors in their management and maintenance. Studying their participation in shelterbelt conservation activities and the factors influencing it holds significant reference value for achieving long-term, sustainable management of these shelterbelts. Existing research on farmer participation in shelterbelt management often logically begins by examining their willingness to participate. However, when engaging in collective management actions with quasi-public goods attributes, farmers’ willingness does not always translate into actual behavior, and a divergence between the two may occur ^11,12^. Therefore, investigating the factors influencing farmers’ willingness and behavior in participating in shelterbelt management, analyzing the differences and intrinsic connections between the two, and subsequently promoting the transformation of farmers’ willingness into action are crucial for achieving long-term shelterbelt management. Farmers’ participation in shelterbelt management is simultaneously influenced by internal factors and external environmental conditions^13^. Among internal factors, farmers’ cognition serves as the precursor to production decisions and the foundation for the formation of behavioral intentions. Existing research on farmers’ perceptions of shelterbelts has largely focused on ecological knowledge, examining how farmers’ understanding of ecological functions influences their willingness or behavior to participate^2,12,14^. However, this body of work has overlooked the potential impact of deeper ecological cognition, such as ecological responsibility and ecological values, on farmers’ participation in shelterbelt management. However, individual factors such as knowledge, ecological responsibility, and ecological values may all influence behavioral choices. Existing research indicates that deeper cognitive elements like ecological values and ecological responsibility significantly and positively affect farmers’ ecological conservation behaviors^15–17^.Meanwhile, as afforestation and greening projects continue to advance, ecological environments have significantly improved, leading to a decline in public attention toward farmland shelterbelts^6,9^. Field research reveals that some farmers hold distorted perceptions regarding their responsibilities for shelterbelt management, tending to externalize accountability to government agencies, village committees, and other relevant organizations. This tendency to externalize responsibility significantly diminishes their willingness to participate in management activities. Therefore, focusing solely on farmers’ ecological knowledge through a unidimensional analysis fails to comprehensively explain or effectively address the current reality of insufficient farmer participation in shelterbelt management. Organizational support serves as a crucial external factor in promoting farmer participation in collective public affairs^18,19^. Farmland shelterbelts possess typical public goods attributes, and their effective management relies both on the collaborative participation of farmers and the support and coordination provided by village organizations. However, existing research has explored the role of organizational support in farmers 'ecological participation behaviors, including participation in the maintenance of small-scale farmland water conservancy facilities, adoption of conservation tillage techniques, and rural living environment improvement^18–21^. Yet studies focusing on farmland shelterbelt maintenance remain limited. Even in the few existing studies on farmland shelterbelt maintenance, few integrate farmers’ intrinsic ecological awareness with organizational support from external environments into a unified analytical framework making it challenging to systematically reveal the shared driving mechanisms of these two factors in farmers’ willingness and behavior regarding shelterbelt maintenance. The participatory development perspective emphasizes that the full involvement of target groups throughout the entire project lifecycle—including planning, implementation, and monitoring—is a core element for project success^22^. This aligns with Li’s definition of farmer participation as an active process whereby farmers exert positive influence on the implementation and direction of rural development projects by engaging in project decision-making, execution, and monitoring/evaluation^23^. Furthermore, Qiao^24^ categorizes farmer participation in farmland water conservancy facility construction into three modes: decision-making, action, and feedback. Dong et al.^25^ explore farmer participation in rural ecological and environmental governance by examining three dimensions: participation in ecological and environmental decision-making, participation in ecological and environmental protection activities, and participation in ecological and environmental supervision. Similarly, the routine management of shelterbelts encompasses the entire process from shelterbelt management decision-making and routine supervision to collective management actions.

Existing research provides a theoretical foundation for this paper, but further exploration can be pursued in three dimensions. First, current studies on shelterbelt management predominantly focus on macro-level aspects such as management effectiveness, challenges, and models^6,10,26,27^. Research analyzing shelterbelt management behaviors from the micro-level perspective of farmers requires deeper investigation. Second, regarding farmers’ cognitive dimensions toward shelterbelts, most studies emphasize perceptions of ecological efficacy while neglecting deeper psychological drivers such as ecological responsibility and values. Third, existing research on farmer participation in shelterbelt management primarily examines single dimensions like collective management activities^5^, lacking a comprehensive analysis of farmer involvement in shelterbelt management. Future research should integrate farmers into the entire chain of management—from plan formulation and oversight to collective management activities. This paper’s potential contribution lies in integrating Elinor Ostrom’s^28^ Social-Ecological System (SES) theoretical framework with interactive determinism to systematically analyze how ecological cognition, organizational support, and their interactions influence farmers’ willingness and behavior toward participating in shelterbelt management. Furthermore, this study categorizes farmer participation willingness and behavior into three dimensions: “participation in formulating shelterbelt management plans,” “participation in supervising the implementation of shelterbelt management,” and “participation in collective shelterbelt management activities.” This approach overcomes the limitations of previous studies that examined only a single behavioral dimension, revealing more comprehensively the holistic process of farmer participation in shelterbelt management. This study focuses on southern Xinjiang, a typical forest-fruit cultivation region. Given the extensive scale of the local forest-fruit industry, where fruit trees have become the primary protected objects within the farmland shelterbelt network^29^, the research centers on forest-fruit farming households. By deeply exploring the factors influencing their willingness and behavior in participation, this study holds significant theoretical value and practical implications for revealing the micro-level mechanisms of sustainable shelterbelt management in southern Xinjiang.

## Theoretical framework and research hypotheses

### Theoretical framework

**Fig. 1:**
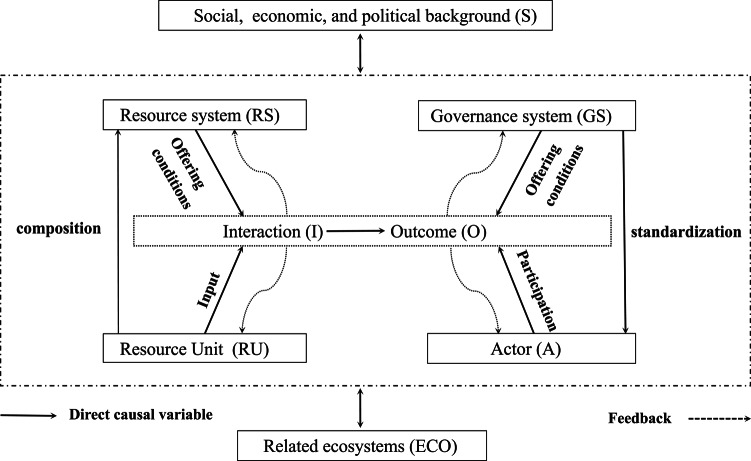
Conceptual diagram of the socio-ecological system framework.

The core function of shelterbelt management is to maintain the stability of the “ecological forest-economic forest” composite system. Within this composite system, ecological forests, economic forests, and farmers form an interdependent organic whole. The system’s overall functionality relies on the coordinated operation of all elements, with farmers’ management practices serving as the crucial link connecting ecological and economic forests—balancing the system’s ecological and economic benefits. Therefore, analyzing the mechanisms influencing farmers’ management intentions and behaviors requires consideration of both the system’s holistic characteristics and the interactive logic among its components. Farmers’ willingness or behavior to participate in shelterbelt management is fundamentally the result of multiple factors interacting within both social and ecological systems^5,30^. The SES framework centers on the holistic interconnection between society and ecosystems, integrating variables such as resource systems, governance systems, resource units, and users to form a multidimensional, coupled, and interactive organism. This provides a more comprehensive and multidimensional perspective for analyzing the complex mechanism of farmer participation in shelterbelt management. As an integrated analytical tool for public resource governance, the SES framework has been widely applied in studies on rural governance, irrigation systems, forest resources, and other public resource management contexts^31,32^. This framework comprises multi-level variables, allowing researchers to decompose its levels and select variables based on specific research subjects and objectives^33^. The first-level variables comprise eight fundamental components (Fig. 1). The four core subsystems—Resource System (RS), Resource Unit (RU), Governance System (GS), and Actor (A)—collectively influence the interaction process (I) and interaction outcome (O) within the action context. Meanwhile, the interactions among these variables are shaped by subsystems such as the socio-economic-political environment (ECO) and the relevant ecosystem (S). These eight fundamental components can be further decomposed into secondary variables (Table 1) and additional levels. These variables enable more detailed descriptions of various aspects within socio-ecological systems and provide diverse options for studying collective action issues across different contexts.

**Table 1:** Selected Secondary Variables in the SES Framework.

Resource system (RS)	Governance system (GS)	Resource unit (RU)	User (U)
RS1, Resource sector	GS1, Government organizations	RU1,Liquidity of resource unit	U1,Number of users
RS2, Whether the system boundaries are clear	GS2, Non-governmental organizations	RU2,Increase, decrease, and turnover rate	U2,Socio-economic attributes of users
RS3, System size	GS3, Network structure	RU3,Interactivity of resource units	U3,Use history and experience
RS4, Man-made facilities	GS4, Property rights system	RU4,Economic value of resource units	U4,Geographical location
RS5, System productivity	GS5, Operational rules	RU5,Number of units	U5,Leadership
RS6, Ability to maintain self-balance	GS6, Rules of collective choice	RU6,Distinguishable features	U6,Social norms/social capital
RS7, Predictability of facility provision	GS7, Constitutional rules	RU7,Spatiotemporal allocation of resources	U7,Perception of socio-ecological systems
RS8, Resource storage feature	GS8, Surveillance and sanctions rules		U8, Dependence on resources
RS9, Locations			U9. Techniques used
Interaction (I) → Outcomes (O)			
I1, Level of resources obtained	I5, Investment in equipment maintenance	I9,Supervision activities	O1, Social performance measurement
I2, Information sharing between users	I6, Lobbying behavior	I10,Evaluation activities	O2,Ecological performance measurement
I3, Negotiation	I7, Self-organizing actions		O3,Externalities
I4, Conflicts between users	I8, Networked action		

This paper draws upon WangYahua’s^33^ discussion of key variables within the SES framework. Building upon existing relevant research, it decomposes these variables into third-level components based on three primary variables: resource systems, governance systems, and actors. Farmers’ ecological cognition refers to their holistic understanding of the surrounding ecological environment and their knowledge and mastery of ecological science^34^. It is the primary factor constraining their decision-making behavior, and their participation in shelterbelt management is essentially a conscious action based on effective cognition. As key actors in shelterbelt management, farmers’ ecological cognition levels directly influence their participation in management activities. Ecological cognition can be assessed across three dimensions: ecological knowledge, ecological values, and ecological responsibility^35^. Therefore, these three indicators are incorporated into the knowledge (U7) component of the socio-ecological system within the actor (U) subsystem. In the context of farmland shelterbelt management, organizational support refers to the level of support provided by the farmer’s village collective and government for their participation. This helps elucidate the relationship between farmers and village/government organizations within an “embedded” social structure. Ling et al.^36^ have categorized organizational support into two dimensions: affective support and instrumental support. Thus, both instrumental and affective support under the organizational support dimension should be incorporated into the operational rules of the government organization (GS-1) within the governance system (GS). Within the SES framework, changes in rural governance systems influence decision-making and behavior by altering the action contexts of users, ultimately affecting outcomes (O)^32^. Farmers’ decisions to participate in shelterbelt management, their willingness to engage in oversight, and their collective management behaviors inherently possess characteristics of collective action. Furthermore, the Theory of Planned Behavior indicates that individual intentions influence actions, and strengthened intentions facilitate behavioral implementation. Based on this, this study defines farmers’ intentions and behaviors regarding shelterbelt management participation as a measure of social performance. Additionally, the growth status of shelterbelts directly relates to their protective benefits. This study incorporates the growth status of shelterbelts as a key indicator for measuring resource system productivity (RS5) into the analysis of control variables.

Building upon this foundation, Albert Bandura’s “interactionist determinism” further supplements the micro-level dynamic interaction logic of “individual-environment-behavior.” A core tenet of this theory is that behavior is not driven by a single factor but emerges from the interplay among individuals, environments, and behaviors^37^. This study places the core variables within the SES framework within the explanatory logic of reciprocal determinism. Ecological awareness within the actor subsystem constitutes the individual’s internal conditions; organizational support within the governance subsystem forms the critical external environment, while farmers’ participation willingness and behavior serve as the outcomes to be explained. Based on this integrated framework, this paper systematically analyzes how ecological cognition, organizational support, and their interaction terms influence farmers’ willingness and behavior to participate in shelterbelt management. This approach establishes an organic connection between macro-level structures and micro-level mechanisms by examining the interplay among internal factors, external environments, and behaviors.

## Research hypotheses

### The impact of farmers’ ecological awareness on their participation intentions and behaviors

In this paper, ecological cognition encompasses three progressively deepening dimensions. Ecological knowledge serves as the foundation for ecological cognition formation, focusing on farmers’ understanding of the ecosystem service functions provided by farmland shelterbelts. Ecological responsibility reflects farmers’ recognition of their stewardship duties, built upon ecological knowledge. Ecological values represent the synthesis of ecological knowledge and responsibility awareness, embodying farmers’ recognition of the alignment between shelterbelts and local ecological sustainability. Farmers’ recognition of the ecosystem service value of natural resources promotes their willingness or behavior to participate in ecological conservation^1,38^. The process of farmer participation in shelterbelt management is inherently one of improving and enhancing the level of ecosystem services provided by shelterbelts^39^. Farmers with higher recognition of the ecological value of shelterbelts can more comprehensively evaluate the long-term benefits of effective shelterbelt management. For instance, they recognize how the ecological functions of shelterbelts reduce agricultural production risks such as sandstorms or frost damage. They may also prioritize these long-term benefits over the short-term time or labor costs required for immediate participation in management. This rational assessment helps overcome the “free-riding” mentality that often hinders collective action among farmers. From the perspective of collective action theory, public demand for public goods constitutes the core driving force behind collective action^40^. When farmers recognize through ecological knowledge that the ecological value of shelterbelts is closely linked to their production and livelihoods, they will enhance their awareness of stewardship responsibilities and willingness to participate based on self-interest considerations, thereby generating positive effects on actual participation behaviors. Furthermore, as farmers accumulate and deepen their ecological knowledge, their ecological responsibility consciousness is stimulated, forming a stable and conscious ecological value system that provides a stable endogenous motivation for their participation in stewardship behaviors^16,17^. Secondly, as a key psychological variable influencing pro-environmental behavior, ecological responsibility effectively stimulates farmers’ altruism^41^ and positively impacts their ecological conservation actions^17^. In ecologically fragile regions, farmers’ ecological rationality often surpasses their economic rationality^2,12^. This endogenous sense of responsibility and identity serves as a powerful driving force, motivating them to transcend personal economic rationality and engage in collective affairs. Finally, ecological values, as a deeper psychological orientation, provide further stable support for the transformation process of conservation behaviors^16^. Ecological values profoundly shape farmers’ value judgment systems, thereby influencing their environmental behaviors^15^. Farmers with positive ecological values are more likely to consciously integrate ecological conservation goals into their behavioral decisions, leading to more standardized implementation of ecological conservation practices such as shelterbelt management. Based on this, Research Hypothesis 1 is proposed.

#### Hypothesis 1

Ecological cognition exerts a significant positive influence on farmers’ willingness and behavior to participate in shelterbelt management.

#### Hypothesis 1a

Ecological knowledge exerts a significant positive influence on farmers’ willingness and behavior to participate in shelterbelt management.

#### Hypothesis 1b

Ecological responsibility significantly and positively influences farmers’ willingness and behavior toward participating in shelterbelt management.

#### Hypothesis 1c

Ecological values significantly and positively influence farmers’ willingness and behavior toward participating in shelterbelt management.

### Impact of organizational support on farmers’ participation intentions and behavior

Organizational support is a key external factor driving farmers’ behavioral decisions. It describes members’ overall perception of whether their organization values their contributions or cares about them. Organizational support theory posits that when members perceive emotional or material support from their organization, their sense of responsibility toward it increases, prompting them to proactively strive for the organization’s common interests^19^. The emotional support provided by the village collective enables farmers to perceive the organization’s care and respect for their labor. This directly influences farmers’ psychological satisfaction, making them feel obligated to reciprocate and contribute to the organization. Consequently, it enhances their attachment to the organization, stimulates their subjective initiative, and motivates them to respond to the organization’s support with reciprocal behaviors such as active participation in shelterbelt management. Moreover, emotional support signals to farmers their importance in conservation efforts and the organization’s commitment to shelterbelt management. This communication enhances farmers’ self-esteem and group identity, thereby increasing their willingness and actions to participate in collective public affairs. Advancing participatory governance in ecological conservation requires full information disclosure as a prerequisite to guide and empower public oversight and evaluation^42^. Information asymmetry is also a primary driver of irrational behavior among farmers. As representatives of formal institutions, village collectives provide authoritative and systematic information on shelterbelt management through instrumental support. This effectively reduces information asymmetry within farmer groups, promoting effective oversight and mutual cooperation in the management process. Furthermore, the management and operation of farmland shelterbelts require certain silvicultural techniques^2^. The forestry technical training provided by village collectives can help farmers more comprehensively master management techniques, effectively alleviate management pressures related to technical application, and provide safeguards for the long-term management of shelterbelts. Based on the above analysis, Research Hypothesis 2 is proposed.

#### Hypothesis 2

Organizational support exerts a significant positive influence on farmers’ willingness and behavior to participate in shelterbelt management.

#### Hypothesis 2a

Instrumental support exerts a significant positive influence on farmers’ willingness and behavior to participate in shelterbelt management.

#### Hypothesis 2b

Emotional support exerts a significant positive influence on farmers’ willingness and behavior to participate in shelterbelt management.

### The interactive effects of ecological awareness and organizational support on farmers’ participation intentions and behaviors

**Fig. 2:**
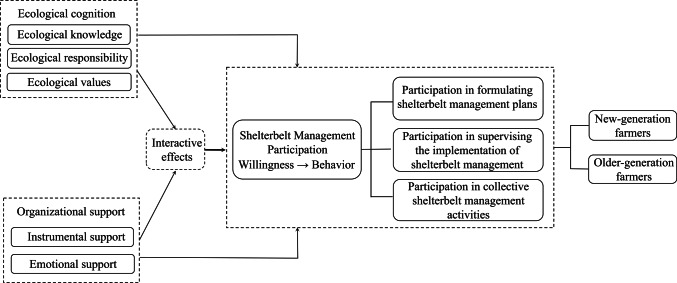
Theoretical framework of ecological cognition and organizational support influencing farmers’ participation intentions and behaviors.

According to interaction determinism, internal conditions, external environments, and subject behaviors interact and interconnect to ultimately become the key forces driving endogenous development among farming households^16^. Among the influences of internal conditions and behavioral agency, an individual’s ecological cognition serves as a latent factor shaping behavioral emergence. While this cognition motivates and sustains behavioral development, it may remain constrained by internal or external factors, hindering its translation into actual participatory actions. Regarding the influence of external environment and subject behavior, government-provided organizational support expands farmers’ behavioral options and enhances action feasibility through positive empowerment, thereby affecting the occurrence, implementation intensity, and sustained stability of subject behavior. That is, ecological cognition and organizational support exhibit an interactive effect on farmers’ willingness and behavior to participate in shelterbelt management, mediated through a regulatory mechanism. Specifically, tool support provided by village collectives—such as offering protective forest management information or technical training—reduces farmers’ information search costs and technical barriers to participation. This effectively amplifies the driving effect of ecological cognition on actual participation, enabling cognitively aware farmers to more readily implement concrete actions. Emotional support, meanwhile, strengthens the intrinsic motivation for translating cognition into action by enhancing farmers’ psychological sense of belonging and responsibility. Based on this, Research Hypothesis 3 is proposed. (Fig. 2).

#### Hypothesis 3

Organizational support positively moderates the positive effect of farmers’ ecological cognition on their participation willingness and behavior.

#### Hypothesis 3a

Instrumental support positively moderates the positive effect of farmers’ ecological knowledge, ecological responsibility, and ecological values on their participation willingness and behavior.

#### Hypothesis 3b

Emotional support positively moderates the positive effect of farmers’ ecological knowledge, ecological responsibility, and ecological values on their participation willingness and behavior

## Data sources, model construction, and variable definitions

### Data sources

Wensu County, under the jurisdiction of Aksu Prefecture in Xinjiang, is situated in the transitional zone between basins and mountains in the upper reaches of the Tarim River basin. It not only exhibits typical mountain-oasis-desert distribution characteristics but also features a warm temperate continental arid climate. Additionally, it serves as a model region for the “ecological forest-economic forest” composite planting model in southern Xinjiang. Agriculture is the dominant industry in Wensu County, with forestry and fruit cultivation emerging as a crucial pillar for increasing farmers’ income. Whether in terms of natural geography or socio-economic development patterns, Wensu County exhibits strong typicality and representativeness, concentrating the core developmental characteristics of the upper Tarim River basin. It serves as an ideal sample area for relevant regional studies. Therefore, this paper selects Wensu County, with its extensive forestry and fruit cultivation area, as a typical survey region for examining farmer participation in the management and protection of farmland shelterbelts. Data in this paper originate from field research conducted by the research team in Wensu County in July 2025. The survey targeted five townships with substantial orchard cultivation: Kokeyazhen, Topuhan Town, Jiamu Town, Yixilemuqi Township, and Keziler Town. Using random household selection, one-on-one questionnaire surveys were administered, supplemented by in-depth semi-structured interviews. The sample encompassed 15 administrative villages. Questionnaire topics included household characteristics, agricultural production and management conditions, perceptions of the ecosystem service value of shelterbelts, willingness to participate in management and maintenance, and related behaviors. A total of 355 questionnaires were collected. After excluding those with missing or incomplete responses to key indicators, 322 valid questionnaires were retained, achieving a response rate of 90.70%. It is hereby confirmed that all research involving human subjects and the associated methods have been conducted in accordance with relevant guidelines and regulations, and that all protocols have been approved by the Institutional Review Board of Xinjiang Agricultural University. Furthermore, it is confirmed that informed consent has been obtained from all research participants.

From the perspective of individual and production characteristics of respondents, the overall educational attainment of farmers is relatively low, with 84.47% holding a junior high school diploma or lower. Educational disparities within this group are minimal. The average farming tenure is approximately 14 years, and 75.78% of farmers operate on plots of 15 mu (approximately 2.4 acres) or larger. Land parcels are relatively concentrated, and forest land quality is generally good, with 74.22% rated at average or above. Regarding household characteristics, most surveyed households comprised 4–6 members. Purely agricultural or agriculture-dominant income sources accounted for 90.06% of household earnings, with traditional farming serving as the primary income generator. Additionally, 77.02% of households employed 2–5 agricultural laborers.

### Model selection

In this study, farmers’ participation willingness and behavior are treated as binary choice variables, and the Probit model is employed. Since farmers’ participation willingness and behavior are not mutually independent, modeling them separately using Probit models may result in efficiency loss. Therefore, a bivariate Probit regression model is adopted^45^. Both farmer willingness and behavior face a binary decision problem, with four possible interactions between them: (0, 0), (0, 1), (1, 0), and (1, 1). The model is specifically formulated as:

1 $$\left\{ {\begin{array}{*{20}c} {{\mathrm{y}}_{1}^{*} =\upbeta _{1} {\mathrm{x}}_{1} +\upvarepsilon _{1} } \\ {{\mathrm{y}}_{2}^{*} =\upbeta _{2} {\mathrm{x}}_{2} +\upvarepsilon _{2} } \\ \end{array} } \right.$$

Among these, ${\mathrm{y}}_{{1}}^{*}$ and ${\mathrm{y}}_{{2}}^{*}$ represent unobservable latent variables, x_1_ and x_2_ denote the vectors of explanatory variables for farmers’ willingness and behavior to participate in management and protection, respectively, β_1_ and β_2_ are the coefficient vectors to be estimated, while ε_1_ and ε_2_ are random disturbance terms following a bivariate joint normal distribution with a correlation coefficient ρ between them, as follows:

2 $$\left( {\frac{{\upvarepsilon _{1} }}{{\upvarepsilon _{2} }}} \right)\sim {\mathrm{N}}\left\{ {\left( \frac{0}{0} \right),\left( {\frac{{1\uprho }}{{\uprho 1}}} \right)} \right\}$$

When ${\mathrm{y}}_{{1}}^{*}$ > 0, it indicates that farmers have the willingness to participate in management and maintenance; otherwise, ${\mathrm{y}}_{{1}}^{*}$ = 0. Similarly, when ${\mathrm{y}}_{{2}}^{*}$ > 0, it indicates that farmers have the behavior of participating in management and maintenance; otherwise,${\mathrm{y}}_{{2}}^{*}$ = 0. Therefore, the relationship between ${\mathrm{y}}_{{1}}^{*}$ and y_1_, as well as between ${\mathrm{y}}_{{2}}^{*}$ and y_2_, can be established by the following equations:

3 $${\mathrm{y}}_{{1}} \left\{ {\begin{array}{*{20}l} {1,} \hfill & {{\mathrm{y}}_{{1}}^{*} { > 0}} \hfill \\ {0,} \hfill & {{\mathrm{y}}_{{1}}^{*} \le 0} \hfill \\ \end{array} } \right.\quad {\mathrm{y}}_{{2}} \left\{ {\begin{array}{*{20}l} {2,} \hfill & {{\mathrm{y}}_{{2}}^{*} { > 0}} \hfill \\ {0,} \hfill & {{\mathrm{y}}_{{2}}^{*} \le 0} \hfill \\ \end{array} } \right.$$

The only connection between the two equations is the correlation between the random disturbances *ε*_*1*_ and *ε*_*2*_. Testing the null hypothesis “H0:ρ = 0” determines the necessity of model selection. Ifρ = 0, the bivariate Probit model is equivalent to two separate Probit models. Ifρ ≠ 0, correlation exists between ${\mathrm{y}}_{{1}}^{*}$ and ${\mathrm{y}}_{{2}}^{*}$, necessitating the use of a bivariate Probit model. Specifically, ρ > 0 indicates a complementary relationship between the two variables, while ρ < 0 indicates a substitution relationship.

### Variable definitions

1. Dependent variable. This study examines farmers’ participation in shelterbelt management through three dimensions: involvement in formulating the village’s shelterbelt management plan, participation in supervising the implementation of shelterbelt management measures, and engagement in collective shelterbelt management activities within the village. For each dimension, we simultaneously assess farmers’ willingness to participate and their actual participation behavior. In the empirical model, y1 uniformly represents “willingness to participate,” and y2 uniformly represents “participation behavior.”

In measuring individual behavioral intentions, the academic community commonly employs direct questioning about willingness^45^. To avoid significant measurement errors (due to social desirability bias stemming from the quasi-public good nature of shelterbelts, which could overestimate participation willingness) in dichotomous variables, this study adopts a five-point Likert scale ranging from “very willing” to “very unwilling.” Survey results indicate that among the interviewed farmers, the cumulative proportions expressing “very willing” and “willing” to participate in the formulation of shelterbelt management plans, the implementation and supervision of management activities, and collective management actions were 67.39%, 51.86%, and 50.31%, respectively. Further analysis reveals that actual participation patterns among surveyed farmers exhibit a characteristic of “higher engagement in practical activities than in plan formulation and supervision.” Specifically, the highest proportion of farmers (67.7%) participated in collective shelterbelt management activities, indicating a preference for collective management behaviors directly linked to their production interests and with stronger operational feasibility. Such activities often directly improve agricultural production environments, making the perceived effects of participation more tangible and thus generating higher enthusiasm among farmers. In contrast, the proportions of households participating in the formulation of shelterbelt management plans and the supervision of management implementation were 51.55% and 47.2%, respectively. This reflects that the level of participation in management decision-making and supervision remains relatively limited.

(2) Key explanatory variables. Ecological cognition encompasses ecological knowledge, ecological responsibility, and ecological values. Regarding ecological knowledge, farmers’ understanding of the ecosystem service functions of shelterbelts was measured using indicators adapted from Zhang et al.^14^ and Zhang et al.^39^ on farmers’ perceptions of shelterbelt ecosystem services in arid regions, combined with the actual agricultural production context of the study area. This study selected the following five ecosystem service functions to explore farmers’ awareness levels: windbreak and sand fixation, microclimate improvement, water conservation, soil improvement, and increased crop yields. Ecological responsibility was measured based on the statement “Participating in shelterbelt management is every farmer’s responsibility.” Ecological values were assessed through two items: “The mixed planting model of ecological forests (shelterbelts) and economic forests (fruit trees) aligns with local ecological sustainability requirements” and “In the long run, the ecological protective value of shelterbelts outweighs the yield losses caused by their negative edge effect. ”Organizational support was measured by tool-supported variables, drawing from Wen et al.^2^ and Yang et al.^18^. The tool support variable was measured across two dimensions: “Village organizations provide comprehensive information on shelterbelt management” and “Village organizations offer technical training on afforestation and forest protection.” The affective variable was assessed through two dimensions: “Village organizations value your contributions to shelterbelt management” and “Village organizations respond promptly to your suggestions and feedback.” For each item, respondents’ responses ranging from “Strongly Disagree” to “Strongly Agree” were assigned integer values from 1 to 5, respectively.
(3) Control Variables. In the empirical analysis, this study selected control variables from the secondary variables of actors and resource systems within the SES framework. These include characteristics of individual farmers and household agricultural production, as well as the current status of shelterbelt production, to ensure that potential changes in related subsystems are controlled when analyzing the impact of farmers’ ecological cognition and organizational support on their participation in shelterbelt management and conservation activities. Among these, individual actor characteristics encompass age, educational attainment, and years of farming experience; household agricultural production characteristics encompass cultivated area, forest plot fragmentation, share of agricultural income, forest land quality, and number of agricultural laborers. For assessing the current growth status of shelterbelt strips within the resource system, this study adopts a comprehensive evaluation framework from Zhang et al.^14^, considering dimensions such as growth condition, structural integrity, pest and disease incidence, and irrigation water security.
(4) To objectively reflect evaluation indicators such as ecological awareness, organizational support, and current growth status of shelterbelts, the entropy weighting method was applied to assign weights to indicators with multiple items. Comprehensive scores were calculated and used as dimension-specific indicators. Variable selection, definitions, values, and descriptive statistics are presented in Table 2.

**Table 2:** Variable definitions and assignments.

Variable type	Variable Name	Variable Definition	Variable Assignment	Mean	Standard deviation
Dependent variable	Participation Intent	Participation in formulating shelterbelt management plans	Very unwilling = 1; Unwilling = 2; Neutral = 3; Willing = 4; Very willing = 5	3.556	1.398
		Participate in the supervision of shelterbelt management implementation		3.386	1.464
		Participate in collective shelterbelt management activities		3.199	1.429
	Participation Behavior	Participation in formulating shelterbelt management plans	1 = Yes, 0 = No	0.516	0.500
		Participate in the supervision of shelterbelt management implementation		0.481	0.536
		Participate in collective shelterbelt management activities		0.677	0.468
Core explanatory variable	Ecological cognition	Ecological knowledge	Calculated using the entropy value method	3.178	0.911
		Ecological responsibility	Strongly disagree = 1; Disagree = 2; Neutral = 3; Agree = 4; Strongly agree = 5	3.957	1.196
		Ecological values	Calculated using the entropy value method	3.344	0.958
	Organizational support	Instrumental support	Calculated using the entropy value method	3.029	1.225
		Emotional support	Calculated using the entropy value method	3.320	1.070
Control variables	Resource System	Current Growth Status of Shelterbelt	Calculated using the entropy value method	3.083	0.911
	Farmers’ Individual Characteristics	Age	18 ~ 30 = 1; 31 ~ 40 = 2; 41 ~ 50 = 3; 51 ~ 60 = 4; ≥ 60 = 5	3.311	1.212
		Level of Education	Elementary school and below = 1; Junior high school = 2; High school and technical secondary school = 3; Junior college = 4; Bachelor’s degree and above = 5	1.898	0.844
	Characteristics of Agricultural Production	Planting area / hm^2^	0 ~ 1 = 1; 1 ~ 2 = 2;2 ~ 3 = 3; 3 ~ 4 = 4; ≥ 4 = 5	2.354	1.136
		Years of Cultivation	Continuous variable	14.481	7.768
		Number of Family Farm Laborers	Continuous variable	1.966	0.785
		Percentage of Household Income from Farming	≤ 30% = 1; 30% ~ 50% = 2; 51% ~ 70% = 3; 71% ~ 90% = 4; ≥ 90% = 5	3.736	1.172
		Forest Land Quality	Very poor = 1; Poor = 2; Fair = 3; Good = 4; Excellent = 5	3.028	0.868
		Land parcel dispersion	Clustered together = 1; Relatively close = 2; Relatively distant = 3	1.624	0.695

## Results and analysis

### Collinearity test

Prior to regression analysis, to avoid estimation bias caused by multicollinearity among variables, the variance inflation factor (VIF) was calculated for all variables involved. The maximum VIF was 1.87, the minimum was 1.05, and the average was 1.38. All three values were less than 3, indicating no multicollinearity issues among the variables.

### Regression results and analysis

Using Stata software, we estimated a bivariate Probit regression model. Whether it concerns decision-making and supervision in shelterbelt management or collective management behavior, this study concludes that ecological cognition and organizational support exert either positive or negative effects on promoting farmers’ participation willingness and behavior whenever either dimension significantly influences any of these dimensions.

The Impact of Ecological Awareness. As shown in Table 3, ecological knowledge exerts a significant positive influence on farmers’ decision-making regarding participation in shelterbelt management, their willingness to engage in oversight and collective management, and their actual participation behaviors. These effects pass significance tests at both the 1% and 5% levels, confirming Hypothesis 1a. As a core element of farmers’ ecological cognition structure, ecological knowledge not only enhances their understanding of the long-term ecological benefits of shelterbelts but also prompts farmers to engage in rational trade-offs oriented toward long-term interests, reducing their tendency toward free-riding. The higher farmers’ recognition of the importance and ecological benefits of farmland shelterbelts, the stronger their willingness to participate in shelterbelt management^2^, and the greater the alignment between their intentions and actual behaviors^1^. Furthermore, recognizing the value of ecosystem services can stimulate farmers’ ecological responsibility awareness, making them more inclined to comply with collective action norms and thus exhibit significant behavioral responses. Ecological responsibility exerts a significant positive influence on farmers’ willingness to participate in management and their involvement in management decision-making, both passing the 1% significance level test. However, while ecological responsibility significantly positively influences farmers’ decision-making participation behavior, its effect on supervision and collective management behavior is insignificant. This may stem from farmers with strong ecological responsibility having a clearer understanding of their roles. They express their demands through decision-making participation to ensure the rationality of management plans, whereas their involvement in supervision or collective management may be constrained by external institutional environments or limitations in their own labor capacity and time resources. These constraints collectively generate high coordination costs, making it difficult to overcome the inherent free-riding mentality and coordination dilemmas in collective action solely through individual ecological responsibility. Hypothesis 1b is thus confirmed. Ecological values exert a significant negative influence on farmers’ willingness and behavior in participating in management decisions and supervisory activities, failing to confirm Hypothesis 1c. This finding appears to contradict the conventional wisdom that values promote pro-environmental behavior, but it must be understood in light of this study’s contextualized measurement of ecological values. In the measurement, ecological values were defined as a combination of “ideological ecological identity” and “the practical trade-off between ecological value and economic loss.” The negative impact observed in the regression results actually reflects the following: when farmers, after weighing long-term ecological value against the short-term loss of agricultural yield, tend to view economic losses as the dominant factor, their composite ecological value scores are lower, leading to lower levels of management participation. Although as many as 61.49% of the surveyed farmers agreed in Item 1 (purely conceptual level) that the ecological value of shelterbelts aligns with local ecological sustainability, their perception of the economic losses caused by the “land-constraint effect” is more immediate. Based on loss aversion theory, an individual’s perception of lost value is approximately twice that of a gain of the same magnitude, this leads farmers to assign greater weight to economic losses in their decision-making, thereby weakening the positive behavioral motivation that ecological values might otherwise generate. Field research revealed that as the ecological environment in the study area improved and economic tree species such as walnuts and jujubes matured, the windbreak benefits of these stands gradually increased, while some farmers’ recognition of the protective benefits of the shelterbelt further diminished. Furthermore, as the age of the shelterbelt trees increases year by year, the competition for land resources from their root systems and canopies intensifies. The competition for water and nutrients between the shelterbelt and adjacent fruit trees, as well as the shading effect of the shelterbelt, has become more pronounced. Against this backdrop, as many as 39.44% of the surveyed farmers believe that the competition for land resources has surpassed the protective benefits, while only 29.81% believe that the protective benefits remain dominant. This widespread, economically driven cognitive bias causes farmers to prioritize short-term economic gains in their overall cost–benefit analysis, thereby dampening their participation in forest management. This finding aligns with the core logic of the SES framework, where actors’ perceptions are embedded in the concrete characteristics of specific resource systems, and it also supports the theoretical proposition of interactive determinism, which posits that individual cognition continuously interacts with the external environment. However, this finding differs from the research of Cheng et al.^12^, whose studies were conducted during the early, ecologically fragile stages when farmers urgently needed the core protective functions of shelterbelts—such as windbreak and sand fixation, and soil improvement—leading to management decisions characterized by “survival rationality” over “economic rationality.” As the ecological environment in the study area gradually improved, some farmers reduced their emphasis on shelterbelts, shifting their decision-making logic toward economic rationality. The above analysis demonstrates that the three dimensions of ecological cognition exert significantly heterogeneous effects on farmers’ willingness and behavior toward management and maintenance, partially supporting Hypothesis 1.

**Table 3:** Effects of ecological awareness and organizational support on farmers’ participation intentions and behaviors.

Variable name	Participate in plan formulation		Participate in monitoring implementation		Participate in collective management and protection	
	y1	y2	y1	y2	y1	y2
Ecological knowledge	0.270**	0.291**	0.256**	0.553***	0.265**	0.315***
	(0.106)	(0.119)	(0.116)	(0.119)	(0.115)	(0.113)
Ecological responsibility	0.208***	0.301***	0.267***	0.076	0.467***	0.103
	(0.077)	(0.086)	(0.095)	(0.080)	(0.106)	(0.070)
Ecological values	− 0.168*	− 0.276***	0.012	− 0.298***	0.005	− 0.138
	(0.095)	(0.100)	(0.105)	(0.107)	(0.121)	(0.094)
Instrumental support	0.213**	0.342***	0.343***	0.314***	0.114	0.326***
	(0.085)	(0.089)	(0.095)	(0.090)	(0.092)	(0.086)
Emotional support	0.145	0.235**	0.183*	0.061	0.304***	0.078
	(0.097)	(0.102)	(0.104)	(0.104)	(0.107)	(0.097)
Current Status of Shelterbelts	0.114	0.577***	0.138	0.430***	0.154	0.120
	(0.104)	(0.138)	(0.104)	(0.130)	(0.124)	(0.109)
Age	− 0.014	− 0.305***	0.200**	− 0.044	0.192**	− 0.351***
	(0.087)	(0.096)	(0.091)	(0.099)	(0.095)	(0.095)
Level of education	− 0.156	− 0.099	0.139	− 0.113	0.015	− 0.052
	(0.100)	(0.107)	(0.108)	(0.112)	(0.114)	(0.119)
Cultivated area	0.068	− 0.076	− 0.026	0.059	− 0.100	− 0.040
	(0.075)	(0.084)	(0.079)	(0.084)	(0.084)	(0.072)
Years of cultivation	0.015	− 0.010	0.026**	− 0.021*	− 0.002	0.017
	(0.012)	(0.013)	(0.012)	(0.012)	(0.012)	(0.012)
Share of agricultural income	− 0.082	0.006	− 0.017	− 0.062	− 0.077	0.043
	(0.074)	(0.090)	(0.075)	(0.079)	(0.077)	(0.076)
Agricultural labor force	0.268**	0.177	0.190*	0.012	0.277***	0.095
	(0.121)	(0.138)	(0.100)	(0.113)	(0.102)	(0.113)
Forest Land Quality	0.150	− 0.051	0.181*	0.108	0.191*	0.067
	(0.098)	(0.107)	(0.108)	(0.099)	(0.102)	(0.114)
Land parcel dispersion	− 0.068	0.158	0.017	0.203	0.218*	0.109
	(0.125)	(0.136)	(0.140)	(0.127)	(0.128)	(0.131)
Constant	− 2.620***	− 3.720***	− 6.072***	− 3.590***	− 6.065***	− 1.525**
	(0.753)	(0.944)	(0.929)	(0.826)	(0.931)	(0.770)
ρ value	0.447***		0.246**		0.282**	
Wald test of ρ = 0	χ^2^(1) = 15.046, *p* < 0.001		χ^2^(1) = 4.824, *p* < 0.05		χ^2^(1) = 6.200, *p* < 0.05	
Log likelihood	− 294.331		− 312.110		− 296.589	
Prob > chi2	0.000					

*, **, and *** indicate significance at the 10%, 5%, and 1% levels, respectively; the numbers in parentheses are standard errors.

The Impact of Organizational Support. Tool support demonstrated a significant positive influence on farmers’ willingness to participate in shelterbelt management decisions, their willingness to engage in oversight, and their actual participation behaviors. All these effects passed significance tests at the 5% or 1% level, confirming Hypothesis 2a. As a key variable within the governance system, tool support not only directly stimulated farmers’ willingness to participate but also significantly facilitated the transition from intention to action by enhancing the feasibility of their behaviors. Particularly in collective actions with high coordination costs, such as supervision and collective management, tool support becomes an indispensable external enabling factor. Enhancing process transparency and providing technical assistance it helps overcome free-riding tendencies and resolve collective action dilemmas. Emotional support exhibits a significant positive influence on farmers’ willingness to participate in shelterbelt supervision and collective management, as well as their involvement in decision-making processes. Hypothesis 2b is confirmed, passing significance tests at 10%, 1%, and 5% levels, respectively. When organizations place greater emphasis on farmers’ contributions to conservation activities and provide timely responses to their suggestions, farmers perceive increased recognition and respect from the organization. Based on reciprocal behavior patterns and social exchange theory ^19^, farmers become more willing to reciprocate through farmland shelterbelt conservation, fostering a virtuous cycle of interaction. Emotional support did not significantly influence farmers’ monitoring or collective management behaviors. This may stem from the fact that monitoring behaviors may face interpersonal constraints within traditional rural communities, while collective management requires substantial time and labor investment. Although emotional support can enhance willingness by fostering a sense of belonging, translating this support into actual monitoring and collective management actions still necessitates integration with substantive instrumental support. The above analysis demonstrates that organizational support exerts a significant positive influence on farmers’ willingness and behavior toward conservation participation, confirming Hypothesis 2.

Controlling for variable effects. The current growth status of shelterbelts within the resource system did not influence farmers’ willingness to participate, suggesting that such willingness may be more strongly driven by factors related to the actor system and governance system. Resource status exerted a significant positive influence on farmers’ participation in management decisions and monitoring behaviors, reaching statistical significance at the 1% level, but did not affect their participation in collective management activities. The growth condition of shelterbelts serves as crucial external feedback for farmers’ perception of conservation necessity. Field surveys and farmer interviews revealed that shelterbelt tree species in the study area’s townships are predominantly single-species poplar, featuring simple stand structures with weak stress resistance. These stands are susceptible to pest and disease infestations and exhibit relatively high irrigation water consumption^9^. Furthermore, pest and disease outbreaks in shelterbelts not only spread to surrounding orchards but also pose significant challenges for control due to the trees’ height. This not only reduces farmers’ self-management effectiveness but also increases the cost and difficulty of pest and disease control in orchards. To mitigate economic losses and improve pest control outcomes, farmers become more proactive in participating in management plan formulation to voice their demands and engage in daily supervision to ensure implementation. Thus, current growth conditions positively drive both behaviors. Farmers’ age significantly and positively influences their willingness to supervise shelterbelt maintenance and engage in collective management at the 5% significance level. However, it negatively impacts participation in management decisions and collective actions at the 1% level. This may stem from older farmers’ limited energy and physical capacity, making it difficult to translate their willingness into concrete actions—ultimately creating a disconnect between intent and behavior. The number of laborers, forest land quality, and plot fragmentation significantly influence farmers’ willingness to participate in management, but have no significant effect on management behavior. Due to the public good nature of farmland shelterbelts, farmers with differing preferences and resource endowments may struggle to form effective cooperation in the short term ^5^, making it difficult to translate their willingness into action.

### The interactive effects of ecological awareness and organizational support on farmers’ participation intentions and behaviors

There may be certain interactive effects between farmers’ ecological cognition and organizational support. This paper decentralized the three dimensions of ecological cognition (ecological knowledge, ecological responsibility, ecological values) and the two dimensions of organizational support (instrumental support, affective support), constructed interaction terms, and incorporated them into regression models for testing. The results are shown in Tables 4 and 5.

**Table 4:** Effects of ecological awareness and tool support interaction on farmers’ participation intentions and behaviors.

Variable name	Participate in plan formulation		Participate in monitoring implementation		Participate in collective management and protection	
	y1	y2	y1	y2	y1	y2
Ecological knowledge	0.373***	0.350***	0.311***	0.581***	0.398***	0.365***
	(0.109)	(0.123)	(0.119)	(0.117)	(0.130)	(0.117)
Ecological values	− 0.108	− 0.195*	0.077	− 0.251**	0.116	− 0.082
	(0.099)	(0.102)	(0.109)	(0.101)	(0.123)	(0.097)
Ecological responsibility	0.255***	0.304***	0.321***	0.099	0.570***	0.056
	(0.089)	(0.092)	(0.091)	(0.083)	(0.104)	(0.080)
Instrumental support	0.348***	0.546***	0.426***	0.349***	0.249***	0.367***
	(0.079)	(0.098)	(0.089)	(0.085)	(0.094)	(0.090)
Ecological knowledge × Instrumental support	0.242***	0.189**	0.142	0.129	0.249**	0.050
	(0.082)	(0.086)	(0.088)	(0.090)	(0.099)	(0.100)
Ecological values × Instrumental support	− 0.068	0.025	− 0.021	0.004	− 0.043	0.073
	(0.070)	(0.087)	(0.080)	(0.080)	(0.091)	(0.072)
Ecological responsibility × Instrumental support	0.040	− 0.171**	0.084	0.029	0.137*	− 0.056
	(0.061)	(0.081)	(0.070)	(0.061)	(0.072)	(0.058)
Constant	− 3.238***	− 4.147***	− 6.125***	− 3.719***	− 6.366***	− 1.503*
	(0.797)	(1.005)	(0.929)	(0.789)	(0.927)	(0.799)
Control variables	Controlled	Controlled	Controlled	Controlled	Controlled	Controlled
ρ value	0.445***		0.233**		0.313***	
Wald test of ρ = 0	χ^2^(1) = 14.481, *p* < 0.001		χ^2^(1) = 4.397, *p* < 0.05		χ^2^(1) = 7.468, *p* < 0.001	
Log likelihood	− 289.709		− 309.561		− 292.463	
Prob > chi2	0.000					

*, **, and *** indicate significance at the 10%, 5%, and 1% levels, respectively; the numbers in parentheses are standard errors.

**Table 5:** Effects of the interaction term between ecological awareness and emotional support on farmers’ participation intentions and behaviors.

Variable name	Participate in plan formulation		Participate in monitoring implementation		Participate in collective management and protection	
	y1	y2	y1	y2	y1	y2
Ecological knowledge	0.331***	0.325***	0.275**	0.579***	0.330***	0.335***
	(0.116)	(0.119)	(0.115)	(0.117)	(0.122)	(0.112)
Ecological values	− 0.176*	− 0.303***	− 0.013	− 0.332***	0.040	− 0.186**
	(0.095)	(0.095)	(0.105)	(0.096)	(0.115)	(0.093)
Ecological responsibility	0.253***	0.291***	0.307***	0.079	0.455***	0.173**
	(0.080)	(0.081)	(0.086)	(0.073)	(0.100)	(0.070)
Emotional support	0.329***	0.472***	0.362***	0.297***	0.433***	0.235**
	(0.089)	(0.097)	(0.094)	(0.092)	(0.117)	(0.093)
Ecological knowledge × Emotional support	0.232**	0.173*	0.099	0.192*	0.252**	− 0.071
	(0.094)	(0.090)	(0.092)	(0.109)	(0.112)	(0.109)
Ecological values × Emotional support	− 0.126	− 0.047	− 0.051	− 0.016	0.041	0.037
	(0.082)	(0.090)	(0.085)	(0.091)	(0.098)	(0.086)
Ecological responsibility × Emotional support	0.101	0.008	0.143**	− 0.074	− 0.059	0.084
	(0.062)	(0.067)	(0.059)	(0.063)	(0.092)	(0.065)
Constant	− 2.962***	− 3.589***	− 5.838***	− 3.502***	− 6.504***	− 1.245
	(0.783)	(0.911)	(0.871)	(0.777)	(0.884)	(0.777)
Control variables	Controlled	Controlled	Controlled	Controlled	Controlled	Controlled
Ρ value	0.476***		0.307***		0.312***	
Wald test of ρ = 0	χ^2^(1) = 17.685, *p* < 0.001		χ^2^(1) = 8.234, *p* < 0.001		χ^2^(1) = 7.677, *p* < 0.001	
Log likelihood	− 296.596		− 317.910		− 297.781	
Prob > chi2	0.000					

*, **, and *** indicate significance at the 10%, 5%, and 1% levels, respectively; the numbers in parentheses are standard errors.

The interaction term between ecological knowledge and instrumental support significantly and positively influenced farmers’ willingness and behavior to participate in conservation plan formulation at the 1% and 5% significance levels. It also significantly and positively influenced willingness to participate in collective conservation at the 5% significance level. This indicates that tool support effectively amplifies the promotional effect of ecological knowledge on farmers’ participation willingness and behavior. Particularly in decision-making and collective management processes, tool support lowers participation barriers by providing information and technical training, thereby enhancing farmers’ ability to translate knowledge into action. The interaction term between ecological responsibility and tool support exhibits a dual moderating effect: it significantly positively influences farmers’ willingness to participate in collective management at the 10% significance level. Collective management of shelterbelts requires certain silvicultural techniques, such as pest control and pruning. When the village collective provides adequate tool support, it effectively reduces high-responsibility farmers’ concerns about participation costs and operational difficulties, enhancing their self-efficacy and perceived behavioral feasibility in completing management tasks. This facilitates the conversion of intrinsic responsibility into explicit participation intentions. However, the interaction term showed a significant negative effect on participation in plan formulation at the 5% level. This result suggests a potential substitution effect in the conversion of ecological responsibility into action: when organizations provide sufficient technical and informational support, farmers become more reliant on guidance and safeguards from these formal entities, partially displacing the driving force of their own sense of responsibility.

The interaction term between ecological knowledge and emotional support passed the 5% and 10% significance tests for willingness to participate in program development and behavioral engagement, respectively. It also passed the 5% and 10% significance tests for participation in monitoring implementation and willingness to engage in collective management, respectively. This indicates that emotional support amplifies the positive impact of ecological knowledge on farmers’ willingness and behavior to participate. By enhancing farmers’ psychological sense of belonging and respect, emotional support motivates them to translate their understanding into concrete actions. The interaction term between ecological responsibility and emotional support showed a significant positive effect at the 5% level on willingness to participate in monitoring implementation, suggesting that emotional support reinforces farmers’ motivation to engage in oversight driven by a sense of responsibility. However, the interaction terms between ecological values and instrumental support/emotional support were insignificant across all dimensions. This indicates that organizational support struggles to moderate ecological values’ influence on farmer behavior, primarily because the “opportunity cost effect”—the perception of economic loss embedded within ecological values—constitutes a deep-seated driver. External support struggles to directly counteract behavioral decisions dominated by economic benefit trade-offs. Thus, Hypotheses 3a and 3b are partially confirmed. The above analysis reveals that organizational support exerts both positive and negative moderating effects on farmers’ ecological cognition, conservation willingness, and behavior. Hypothesis 3 is partially supported.

### Analysis of generational differences in the impact of ecological awareness and organizational support on farmers’ participation intentions and behaviors

With socioeconomic progress and urbanization, generational differentiation among farming households has become increasingly pronounced. New-generation farmers differ from their elders in terms of upbringing, educational attainment, and production methods, potentially leading to divergent perceptions of ecological awareness and organizational support. Consequently, their willingness and behavior toward conservation participation may also vary. Drawing on relevant research^46^, farmers born in 1975 serve as the dividing line: those born before are defined as older-generation farmers, while those born after are classified as new-generation farmers. In the sample, older-generation farmers account for 49.68% of the total, while new-generation farmers constitute 50.31%.

Ecological knowledge exerts a significant positive influence on both the willingness and actual participation of two generations of farmers, though its promotional effect is stronger for the new generation than for the older generation, as shown in Table 6. Supervision significantly and positively influenced both the willingness to participate in collective management and the actual participation behaviors across three dimensions—decision-making, supervision, and collective management—among the new generation of farmers. This may be attributed to their stronger learning and comprehension abilities, coupled with greater exposure to green and environmental education. This not only enables them to grasp the knowledge of the ecosystem services provided by shelterbelts more comprehensively but also allows them to internalize this knowledge as a cognitive framework guiding their own behaviors. Ecological responsibility significantly positively influenced older-generation farmers’ willingness to participate in decision-making and collective management, as well as their actual participation behaviors in these dimensions. However, while it significantly positively influenced the willingness of younger-generation farmers to participate in decision-making, supervision, and collective management, it did not affect their actual participation behaviors in these three dimensions. A possible reason is that younger-generation farmers have a higher degree of diversification in their livelihoods. Increased non-agricultural employment opportunities raise the cost of participating in collective affairs. Higher opportunity costs combined with reduced dependence on agricultural income jointly weaken their motivation to translate responsibility into collective participation. Ecological values exert a significant negative influence on both generations of farmers’ participation in decision-making and oversight activities. As rational smallholders, their actions are driven more by practical considerations of cost–benefit analysis, making it difficult for their conceptual agreement to translate into active participation. Tool support exerts a significant positive influence on both participation willingness and behavior among two generations of farmers, but its promotional effect on participation willingness is more comprehensive for the new generation. Compared to the older generation, the new generation of farmers demonstrates relatively higher responsiveness, comprehension, and acceptance of novel concepts. They can more efficiently absorb and utilize the technologies and information provided by tool support, thereby exhibiting stronger participation willingness. Emotional support significantly and positively influences older farmers’ willingness to participate in supervision and collective management, as well as the decision-making participation of younger farmers. Younger farmers face fewer physical constraints, and emotional support effectively empowers them psychologically, directly driving their decision-making participation. Conversely, older farmers, limited by physical capacity, experience incentives from emotional support that remain largely at the level of willingness.

**Table 6:** Generational differences in farmers’ participation intentions and behaviors regarding ecological awareness and organizational support.

	Participate in plan formulation		Participate in monitoring implementation		Participate in collective management and protection	
	y1	y2	y1	y2	y1	y2
Older-generation farmers						
Ecological knowledge	0.238*	0.172	0.131	0.353**	0.153	0.212
	(0.144)	(0.161)	(0.151)	(0.158)	(0.141)	(0.150)
Ecological responsibility	0.225**	0.627***	0.155	0.069	0.326***	0.202**
	(0.100)	(0.160)	(0.117)	(0.100)	(0.103)	(0.097)
Ecological values	− 0.191	− 0.266**	0.042	− 0.241*	0.018	− 0.147
	(0.120)	(0.135)	(0.124)	(0.136)	(0.130)	(0.114)
Instrumental support	0.070	0.400***	0.251**	0.364***	0.023	0.250**
	(0.107)	(0.115)	(0.110)	(0.114)	(0.103)	(0.102)
Emotional support	0.145	0.185	0.245**	0.100	0.229**	0.135
	(0.111)	(0.126)	(0.114)	(0.125)	(0.114)	(0.116)
Control variables	Controlled	Controlled	Controlled	Controlled	Controlled	Controlled
Prob > chi2	0.000					
New-generation farmers						
Ecological knowledge	0.281	0.331*	0.495**	0.906***	0.539**	0.450**
	(0.178)	(0.187)	(0.202)	(0.200)	(0.262)	(0.191)
Ecological responsibility	0.255*	0.028	0.714***	0.174	1.016***	− 0.119
	(0.137)	(0.123)	(0.164)	(0.129)	(0.197)	(0.124)
Ecological values	0.016	− 0.394**	0.098	− 0.342*	0.146	− 0.044
	(0.158)	(0.183)	(0.169)	(0.182)	(0.230)	(0.181)
Instrumental support	0.590***	0.417**	0.760***	0.394**	0.526**	0.598***
	(0.170)	(0.171)	(0.202)	(0.162)	(0.229)	(0.165)
Emotional support	0.084	0.578***	− 0.348	0.031	0.146	− 0.004
	(0.188)	(0.195)	(0.218)	(0.172)	(0.271)	(0.187)
Control variables	Controlled	Controlled	Controlled	Controlled	Controlled	Controlled
Prob > chi2	0.000					

*, **, and *** indicate significance at the 10%, 5%, and 1% levels, respectively; the numbers in parentheses are standard errors.

### Mediation effect analysis

As shown in Table 3, the correlation coefficients ρ for the bivariate Probit model are all positive and pass the 1% or 5% significance level test. This indicates that there is a certain correlation between farmers’ willingness and behavior to participate in shelterbelt management, with a complementary effect between the two. Specifically, farmers’ willingness to participate in shelterbelt management decisions, supervision, and collective management has a positive effect on their actual participation behavior. As a key external factor, it remains to be further explored whether organizational support indirectly drives behavior by enhancing the willingness to participate, or directly bridges the gap between intention and behavior. The KHB model is a general decomposition method for identifying mediating effects in nonlinear probabilistic models; therefore, this paper employs the KHB model to conduct an analysis of mediating effects. The composite index of organizational support is calculated by summing the values of two variables—instrumental support and emotional support—with equal weights.

As shown in Table 7, willingness to participate plays a significant partial mediating role between organizational support and the formulation of management plans, supervision of implementation, and collective management participation. The mediation effects of each pathway account for 13.18%, 15.57%, and 12.07% of the total effect, respectively. Overall, organizational support primarily influences all three types of management behaviors through direct effects, while the indirect mediating role of farmers’ willingness to participate is also significant. This indicates that organizational support not only directly drives farmer participation in conservation and management but also indirectly promotes the implementation of these behaviors by enhancing farmers’ willingness to participate. Therefore, to address the challenges in farmer participation in shelterbelt conservation and management, it is necessary to focus on fostering willingness while also employing direct measures such as improving participation channels, establishing supervision platforms, and providing technical training and material support. This will strengthen the direct role of organizational support and create a dual-drive mechanism combining tool empowerment and motivation.

**Table 7:** Analysis of the mediating effect of farmers’ willingness to participate on the relationship between organizational support and farmers’ participation behavior.

Mediating effect pathways	Overall effect	Direct effect	Indirect effects	Proportion of the total effect accounted for by the mediating effect
Organizational support → Willingness to participate in policy formulation → Behavior of participating in policy formulation	0.614***(0.107)	0.533***(0.108)	0.081***(0.031)	13.18%
Organizational support → Monitoring the implementation of participation intentions → Monitoring the implementation of participation behaviors	0.398***(0.095)	0.336***(0.098)	0.062*(0.033)	15.57%
Organizational support → Willingness to participate in collective management → Behavior of participating in collective management	0.429*** ( 0.094)	0.377*** ( 0.095)	0.052**(0.026)	12.07%

*, **, and *** indicate significance at the 10%, 5%, and 1% levels, respectively; the numbers in parentheses are standard errors.

### Robustness test

Given that individual behavioral choices are not random occurrences, this study employs the following methods to test the robustness of regression conclusions and validate the reliability of empirical analysis results. (1) Adding control variables. As core participants in village public affairs governance, village cadres possess dual identities as government agents and village leaders. This distinguishes them from ordinary farmers in terms of organizational coordination and information access. Their identity characteristics may potentially influence decision-making, supervision implementation, and collective management willingness and behavior regarding shelterbelt conservation. To further control for potential endogeneity arising from individual identity differences and enhance the robustness of conclusions, this study incorporates the control variable “Are you a village cadre?” (1 = yes, 0 = no) into the original model for testing. Results in Table 8 show that while the magnitude of each variable’s effect varies, the direction of significant variables remains unchanged, thereby further validating the model’s robustness. (2) Variable substitution. To ensure the robustness of regression results, this study tested the robustness by altering variable assignment methods^11^. Given the frequent sandstorms in the study area, farmers demonstrated high awareness of the windbreak and sand fixation functions of shelterbelts at the ecological knowledge level ^39^. This study employed a weighted average method to reconstruct the ecological knowledge dimension’s variable measuring awareness of shelterbelt ecosystem services. This replaced the entropy-based values used in the benchmark regression. As shown in Table 9, the significance and direction of core variable coefficients remained unchanged, confirming the robustness of the regression results.

**Table 8:** Robustness test.

	Participate in plan formulation		Participate in monitoring implementation		Participate in collective management and protection	
	y1	y2	y1	y2	y1	y2
Ecological knowledge	0.256**	0.288**	0.231**	0.542***	0.247**	0.301***
	(0.108)	(0.118)	(0.116)	(0.120)	(0.117)	(0.115)
Ecological responsibility	0.205***	0.300***	0.263***	0.069	0.461***	0.097
	(0.077)	(0.086)	(0.095)	(0.081)	(0.103)	(0.070)
Ecological values	− 0.167*	− 0.275***	0.017	− 0.298***	0.018	− 0.132
	(0.094)	(0.099)	(0.105)	(0.104)	(0.115)	(0.093)
Instrumental support	0.203**	0.339***	0.330***	0.303***	0.098	0.314***
	(0.085)	(0.091)	(0.095)	(0.091)	(0.093)	(0.086)
Emotional support	0.143	0.233**	0.180*	0.053	0.295***	0.069
	(0.096)	(0.101)	(0.104)	(0.105)	(0.106)	(0.097)
Village cadre status	0.427	0.090	0.451	0.514	0.736**	0.430
	(0.321)	(0.332)	(0.275)	(0.347)	(0.356)	(0.302)
Control variables	Controlled	Controlled	Controlled	Controlled	Controlled	Controlled
Constant	− 2.459***	− 3.680***	− 5.878***	− 3.361***	− 5.775***	− 1.319*
	(0.764)	(0.938)	(0.929)	(0.831)	(0.928)	(0.773)
ρ value	0.451***		0.237**		0.273**	
Log likelihood	− 293.338		− 309.491		− 293.078	
Prob > chi2	0.000

**Table 9:** Robustness test.

	Participate in plan formulation		Participate in monitoring implementation		Participate in collective management and protection	
	y1	y2	y1	y2	y1	y2
Ecological knowledge	0.318***	0.323**	0.276**	0.599***	0.266**	0.367***
	(0.114)	(0.126)	(0.122)	(0.123)	(0.119)	(0.125)
Ecological responsibility	0.204***	0.299***	0.265***	0.073	0.470***	0.096
	(0.077)	(0.086)	(0.096)	(0.081)	(0.106)	(0.071)
Ecological values	− 0.163*	− 0.269***	0.021	− 0.284***	0.015	− 0.132
	(0.095)	(0.099)	(0.105)	(0.107)	(0.122)	(0.093)
Instrumental support	0.207**	0.335***	0.338***	0.302***	0.110	0.318***
	(0.085)	(0.089)	(0.095)	(0.091)	(0.092)	(0.086)
Emotional support	0.145	0.234**	0.185*	0.065	0.309***	0.077
	(0.097)	(0.101)	(0.104)	(0.104)	(0.107)	(0.096)
Control variables	Controlled	Controlled	Controlled	Controlled	Controlled	Controlled
Constant	− 2.794***	− 3.839***	− 6.182***	− 3.795***	− 6.142***	− 1.695**
	(0.761)	(0.964)	(0.948)	(0.843)	(0.945)	(0.785)
ρ value	0.442***		0.234**		0.281**	
Log likelihood	-293.521		-311.491		-296.190	
Prob > chi2	0.000					

*, **, and *** indicate significance at the 10%, 5%, and 1% levels, respectively; the numbers in parentheses are standard errors.

## Discussion

This study integrates social and ecological indicator systems based on the SES framework and incorporates interactive determinism to analyze the influence mechanisms of internal factors (ecological cognition) and external environments (organizational support) on farmers’ participation willingness and behaviors across three dimensions—decision-making participation, monitoring actions, and collective management—during their involvement in the conservation of farmland shelterbelts. Findings reveal that among the three dimensions of ecological cognition, ecological knowledge, ecological responsibility, and ecological values exhibit differential impacts on farmer participation. Specifically, at the ecological knowledge level, farmers’ recognition of the ecosystem services provided by shelterbelts promotes their participation willingness to participate and behavior, consistent with existing research by Li et al.^44^ and Zhang et al.^14^. Therefore, enhancing farmers’ ecological knowledge about shelterbelts is crucial for promoting their participation in cooperative management and fully realizing the ecological benefits of these forests. Ecological responsibility also positively influences farmers’ willingness and behavior to participate, consistent with the findings of Feng et al.^17^. Village collectives should focus on cultivating farmers’ ecological responsibility to stimulate their enthusiasm for conservation participation. Ecological values exert a significant negative influence on farmers’ participation willingness and behavior, differing from the findings of Song et al.^16^ and Liu et al.^15^. A possible reason is that, given that farmland shelterbelts possess attributes such as spatial fixity, multifunctionality, and long-term ecological impacts, their protective benefits and negative edge effect inevitably coexist. The ecological values discussed in this paper incorporate a trade-off between the “protective benefits” and “negative edge effect” of shelterbelts. The fact that Hypothesis 1c was not confirmed does not negate the theoretical importance of ecological values. This finding reveals that, in the context of this study, perceptions of economic gains and losses exert a suppressing effect on the behavioral expression of values.Previous studies have indicated that when ecological conservation impacts individual economic interests, farmers exhibit inconsistencies between their environmental values and ecological conservation behaviors^15^,which provides indirect support for this study.Therefore, the relationship between ecological values and pro-environmental behavior may be influenced by specific contextual factors. In areas where the ecological environment has already improved, farmers’ decision-making logic may shift from “survival rationality” to “economic rationality”; in this process, the positive behavioral motivation derived from these values is likely to be undermined by loss aversion. Ruppert D et al.^13^ conducted field research in two regions of Kyrgyzstan in Central Asia—Lake Issyk-Kul and Jalal-Abad—and found that over 59% of surveyed farmers held negative perceptions toward shelterbelts. Yield losses caused by the negative edge effect of shelterbelt strips emerged as one of their primary concerns. Simultaneously, some farmers acknowledged the ecological benefits of shelterbelts yet rejected them due to economic trade-offs. This phenomenon indicates that divergent farmer attitudes stemming from conflicts between ecological value recognition and economic interests are relatively common in cross-regional agroforestry systems. It also implies that governments or village collectives must implement effective measures to offset economic losses caused by the negative edge effect of shelterbelts. This balance requires more than simple economic subsidies; it necessitates establishing a three-dimensional synergistic mechanism integrating technical mitigation, economic compensation, and benefit-sharing. This approach directly addresses farmers’ rational economic demands while gradually guiding the alignment of their ecological values with conservation practices. The analysis reveals that ecological cognition should not be treated as a homogeneous entity when examining farmers’ environmental behaviors. Instead, it requires deconstruction into its multidimensional layers, with close scrutiny of its interaction mechanisms with core variables.

Both instrumental support and emotional support exert a promotional effect on farmers’ willingness and behavior to participate. Studies by Wen et al.^2^, Zhao et al.^19^, and Yang et al.^18^similarly support this empirical finding, further demonstrating the significance of organizational support in driving farmers’ ecological participation behaviors. Ecological knowledge, ecological responsibility, and organizational support exhibit an interactive effect, aligning with the theoretical mechanism of “internal conditions—external environment—subject behavior” being mutually determined. However, organizational support fails to moderate the negative impact of ecological values. While instrumental and affective support can reduce participation costs or enhance psychological identification, they cannot directly compensate for economic losses caused by the negative edge effect of shelterbelts or counterbalance economic trade-offs. Consequently, future policy focus should extend beyond knowledge dissemination and appeals to responsibility, prioritizing the resolution of substantive economic conflicts underlying ecological values.

This study also has certain limitations. First, there is a lack of consideration of endogeneity and a dynamic perspective. In the long term, farmers’ participation in conservation efforts may reinforce the formation of their ecological cognition through practice. However, given the specific context of this study, this reverse causal pathway is not strongly evident in the data. For one thing, ecological cognition is a stable psychological trait that farmers gradually develop through long-term production practices, community culture, and intergenerational transmission. It serves as the psychological foundation and logical starting point for their behavioral decisions, preceding specific conservation participation behaviors in time; thus, short-term conservation behaviors are unlikely to fundamentally alter it. For another, conservation participation by farmers in the study area is predominantly seasonal and responsive collective activities (such as irrigation, pruning, and pest and disease control), with limited depth and frequency of participation. Consequently, it is difficult to generate behavioral feedback of sufficient intensity to reshape deep-seated cognition. Therefore, the causal chain in this study is primarily “cognition drives behavior.” Due to the limitations of cross-sectional data, the above argument cannot entirely rule out the possibility of bidirectional causality. Second, the measurement methods for some variables need improvement. In this study, the indicators measuring farmers’ participation in the formulation of shelterbelt management plans, supervision of implementation, and collective management behaviors are relatively limited and all employ binary variables. This makes it difficult to fully capture the degree of continuity, depth, and varying levels of farmers’ participation; Ecological responsibility is measured using a single item. Although this item directly reflects the core essence of responsibility, it fails to fully capture the multidimensional nature of ecological responsibility, which may to some extent affect the content validity and discriminant validity of the variable measurement. Third, the scope of the study and the dimensions of observation could be expanded. Currently, the study is limited to Wensu County in Xinjiang, with a restricted sample coverage. Furthermore, as the data are cross-sectional, it is difficult to capture the dynamic characteristics of farmers’ management behaviors as they change over time, in response to policy shifts, and in relation to forest stand growth conditions. Consequently, the study falls short in revealing behavioral differences across different regions and among different management entities.

Future research could be further deepened in the following areas: First, by strengthening the identification of causality, future studies could utilize multi-period panel data and combine them with econometric methods such as instrumental variables and lagged-effect models to identify causal relationships more rigorously. Second, measurement tools should be optimized by using multi-level frequency scales to more precisely measure the intensity and frequency of farmers’ participatory behaviors; scales with multiple items and both positive and negative statements should be designed for specific contexts to improve the measurement accuracy and discriminant validity of core variables such as ecological responsibility. Third, expand the scope of research by broadening the survey coverage to include more typical ecologically fragile areas and types of management entities, and extending the study period to capture dynamic changes. Through multi-scale, multi-stakeholder analysis, systematically reveal the complex mechanisms and dynamic evolutionary patterns of farmers’ shelterbelt management behaviors, thereby providing more rigorous and targeted scientific support for the long-term management of farmland shelterbelts in arid regions.

## Conclusions and policy implications

Based on survey data collected in 2025 from 322 farming households in Wensu County, located in the upper reaches of the Tarim River Basin, this study employs a dual-variable Probit regression model within the SES analytical framework, integrated with interactive determinism. It empirically examines the effects and interactions of organizational support at the governance system level and ecological cognition at the actor level on farmers’ willingness and behavior to participate in shelterbelt management. The research also reveals the influence of intergenerational differences among farmers. The findings indicate that: (1) Ecological knowledge significantly enhances farmers’ willingness and behavior across all dimensions of participation through cost–benefit assessment mechanisms. Ecological responsibility, as an intrinsic psychological driver, positively influences participation willingness and plan formulation but shows no significant effect on supervision or collective management due to external institutional constraints and resource limitations. Ecological values exert a significant negative influence on farmers’ willingness to participate in decision-making, decision-making itself, and monitoring behaviors, reflecting the constraints of economic rationality on ecological conservation actions. (2) Organizational support functions as a key external factor with positive effects and differentiated roles. Instrumental support significantly enhances participation willingness and behaviors by reducing information costs and technical barriers. Emotional support positively influences participation in supervision, collective management, and plan formulation primarily by boosting farmers’ sense of belonging and respect, though it requires substantive instrumental support to achieve greater efficacy. (3) Ecological cognition and organizational support exhibit differentiated interactive effects. Tool support enhances the promotional effect of ecological knowledge on participation willingness and behavior while activating the driving force of ecological responsibility toward collective management willingness. However, it exhibits a substitution effect on the conversion of ecological responsibility into decision-making behavior. Emotional support significantly amplifies the positive impact of ecological knowledge and ecological responsibility on participation willingness and behavior. Both types of organizational support fail to moderate the negative effect of ecological values, struggling to offset the economic trade-offs triggered by the negative edge effect of shelterbelts. (4) Intergenerational analysis reveals that younger farmers’ participation willingness and behavior are more influenced by ecological knowledge and tool support, while older farmers rely more heavily on ecological responsibility and tool support.

Based on the above research findings, this paper proposes the following policy implications: (1) Strengthen the cultivation of ecological cognition among farmers to build endogenous motivation for management and protection. Village collectives and relevant departments should prioritize public awareness campaigns on the ecological functions of farmland shelterbelts. This involves conducting social science outreach through channels such as regular village meetings, wall slogans, and WeChat groups. Additionally, ecological knowledge about shelterbelts should be integrated into agricultural technical training systems. Targeted ecological education should be delivered based on local dominant crop structures and ecological vulnerabilities. Concurrently, efforts should focus on cultivating farmers’ ecological responsibility through measures like setting exemplary models and awarding honors to foster a positive atmosphere for forest protection. (2) Addressing the negative edge effect of shelterbelts through multi-dimensional approaches to balance ecological and economic benefits. Government departments continuously promote technical optimizations, such as deepening root-cutting trenches along shelterbelt edges to mitigate the negative edge effect. Furthermore, in subsequent replanting, renewal, and shelterbelt restoration planning, they must consider the windbreak benefits of tall fruit trees in the study area. By optimizing the spatial layout of shelterbelts, they can reduce the land occupied by shelterbelts and the extent of the negative edge effect while ensuring the synergistic ecological functions of both shelterbelts and economic forests, thereby enhancing the economic efficiency of fruit trees^29^^.^ Regarding economic compensation, village organizations should establish reasonable ecological compensation mechanisms, providing subsidies or rent reductions to compensate farmers for losses, thereby boosting their enthusiasm for shelterbelt management. From the perspective of benefit linkage, governments should actively explore carbon credit trading models, distributing a proportion of carbon sink transaction revenues to participating farmers to provide additional income sources. Through carbon credit trading, farmers are incentivized to actively participate in forestry ecological construction, promoting the sustainable development of the ecological-economic forest composite system. (3) Improve organizational support mechanisms to enhance farmer participation effectiveness. Village organizations should build a multidimensional organizational support system centered on “emotional empowerment + tool support.” In the emotional support dimension, provide farmers with ample emotional support through timely responses to their demands and recognition of exemplary cases, thereby strengthening their sense of belonging and identification. On the tool support dimension, first, establish an efficient information management platform for conservation efforts, incorporating a feedback module to enable farmers to promptly report challenges, thereby supporting their participation in monitoring and enhancing their effectiveness. Second, conduct targeted forestry technical training and provide necessary conservation supplies, lowering participation barriers and costs through both technical guidance and material support. This organic integration of tool support and emotional support can collectively guide and encourage farmers to participate more proactively in the daily management of shelterbelts. (4) Implement a differentiated strategy for different generations to precisely meet the needs of farmers across generations. For older generations of farmers, emphasize education on the ecological value of shelterbelts, conveying the significance of management and maintenance in an accessible language. Provide concise, practical technical guidance and information services tailored to their cognitive and behavioral habits. For younger generations, offer efficient, intelligent tool support aligned with their capacity to embrace new technologies. This approach fully stimulates participation willingness while reducing behavioral costs, enabling synergistic efforts across generations in management and maintenance.

## Data Availability

The data that support the findings of this study are available from the corresponding author upon reasonable request.
